# Control of fibrinolytic drug injection via real-time ultrasonic monitoring of blood coagulation

**DOI:** 10.1371/journal.pone.0211646

**Published:** 2019-02-27

**Authors:** Dmitry A. Ivlev, Shakhla N. Shirinli, Konstantin G. Guria, Svetlana G. Uzlova, Georgy Th. Guria

**Affiliations:** 1 National Research Center for Hematology, Moscow, Russia; 2 Moscow Institute of Physics and Technology, Dolgoprudny, Russia; Universiteit Gent, BELGIUM

## Abstract

In the present study, we investigated the capabilities of a novel ultrasonic approach for real-time control of fibrinolysis under flow conditions. Ultrasonic monitoring was performed in a specially designed experimental *in vitro* system. Fibrinolytic agents were automatically injected at ultrasonically determined stages of the blood clotting. The following clots dissolution in the system was investigated by means of ultrasonic monitoring. It was shown, that clots resistance to fibrinolysis significantly increases during the first 5 minutes since the formation of primary micro-clots. The efficiency of clot lysis strongly depends on the concentration of the fibrinolytic agent as well as the delay of its injection moment. The ultrasonic method was able to detect the coagulation at early stages, when timely pharmacological intervention can still prevent the formation of macroscopic clots in the experimental system. This result serves as evidence that ultrasonic methods may provide new opportunities for real-time monitoring and the early pharmacological correction of thrombotic complications in clinical practice.

## Introduction

Monitoring and timely correction of hemostasis is a crucial medical task [1, 2]. A number of severe thrombotic pathologies, such as myocardial infarction and stroke, might occur suddenly and develop very rapidly [3, 4]. In these cases large thrombi occluding blood flow in major arteries can be formed during several minutes [4]. That is why prompt and efficient techniques for hemostasis monitoring are needed.

Over the past two decades turnaround times of clotting tests were substantially reduced by introduction of so-called point-of-care techniques [5]. Novel methods for on-line *ex vivo* monitoring of hemostasis are actively developed [6]. A logical step towards real-time control of hemostasis would be creation of the technique for direct *in vivo* monitoring of intravascular blood coagulation.

One of the possible approaches to creation of such a technique is the use of ultrasonic methods. The idea for applying ultrasonic methods to detect blood coagulation was proposed quite long ago, at first for *in vitro* measurements [7–9]. In recent years, due to developments in modern ultrasonic equipment, this area of research has become active again [10]. Various research teams have offered several ultrasonic techniques for the registration of blood coagulation *in vitro* [11–19]. More recently capabilities of ultrasonic methods for *in vivo* detection of blood coagulation were demonstrated in animal experiments [20–22].

It is essential that ultrasonic methods can detect blood coagulation under flow conditions similar to those that take place in major arteries of human body [23, 24]. This fact reveals the possible application of ultrasonic methods for non-invasive monitoring of coagulation processes in clinical practice [25].

Efficient control of hemostasis implies both its monitoring and means for its pharmacological correction. Usually monitoring can be performed with routine coagulation tests and correction can be achieved by use of anticoagulant drugs [1, 2]. But in acute situations, then formation of arterial thrombi has already started and progress rapidly, coagulation tests are already late and anticoagulants are not capable of thrombi dissolution. In these situations the last line of defense remaining is thrombolytic therapy [26, 27]. Its efficiency drastically depends on the delay after the onset of coagulation processes [28, 29]. A method for real-time monitoring of the onset of intravascular blood coagulation might be very useful in these acute situations for the reduction of onset-to-treatment time.

In the present work we investigated possible benefits of ultrasonic detection of early stages of blood coagulation for fibrinolytic dissolution of forming thrombi. To do so, we designed a special experimental setup for the ultrasonic monitoring of blood coagulation under intensive flow conditions *in vitro*. This setup allowed us to monitor blood coagulation in real time and to perform an automated injection of a fibrinolytic drug at precisely determined stages of the coagulation process. Our experiments showed the following:

1. The ultrasonic method used enables the reliable registration of blood coagulation and following fibrinolytic dissolution of clots. The method facilitates the qualitative evaluation of the efficiency of various fibrinolytic influences and enables the comparison of different fibrinolytic drugs;
2. The fibrinolytic resistance of clots formed under flow conditions increases significantly over the first few minutes of their formation;
3. An immediate injection of a fibrinolytic drug after the ultrasonic registration of the onset of coagulation is able to prevent the formation of large clots in the experimental system.

## Materials and methods

### Ethics statement

This study was approved by the Institutional Committee of Blood Donation and Blood Processing Problems at the National Research Center for Hematology (Permit number: 5/2016). This study was performed with blood received from healthy donors who provided written informed consent before blood collection in accordance with Russian Federal Law No 125 on July 20, 2012. All methods were carried out in accordance with relevant guidelines and regulations (The Order of Russian Health Care Ministry No 183n on April 02, 2014).

### Materials

Whole blood and blood plasma were used in the experiments. The blood and fresh frozen plasma were provided by the Division of Blood and Blood Components Collection and Storage of the National Research Center for Hematology. Blood was preserved in Imuflex (Terumo Europe NV, Belgium) containers with citrate phosphate dextrose (CPD) anticoagulant solution. Plasma was separated from whole blood by centrifugation at 5000 g for 7 minutes.

To initiate coagulation several types of activators were used: 50 μl of 1% kaolin suspension (NPO-Renam, Russia), 50 μl of thromboplastin solution, diluted by 12 times with normal saline (NPO-Renam, Russia) or 10% calcium chloride solution (Mapichem AG, Switzerland). Unless otherwise specified, activation of coagulation was initiated by injection of 600–800 μl of 10% calcium chloride solution.

Three different types of fibrinolytic drugs were used in the experiments: streptokinase (Streptokinaza, Belmedpreparaty, Belarus), tissue-type plasminogen activator (Actilyse, Boehringer Ingelheim International, Germany) and urokinase (Urokinase, Medac GmbH, Germany). The dosages of the fibrinolytic drug were varied in different experiments, while the volume of the fibrinolytic solution injected into the experimental system (0.5 ml) was kept constant.

### Experimental setup

**Fig 1:**
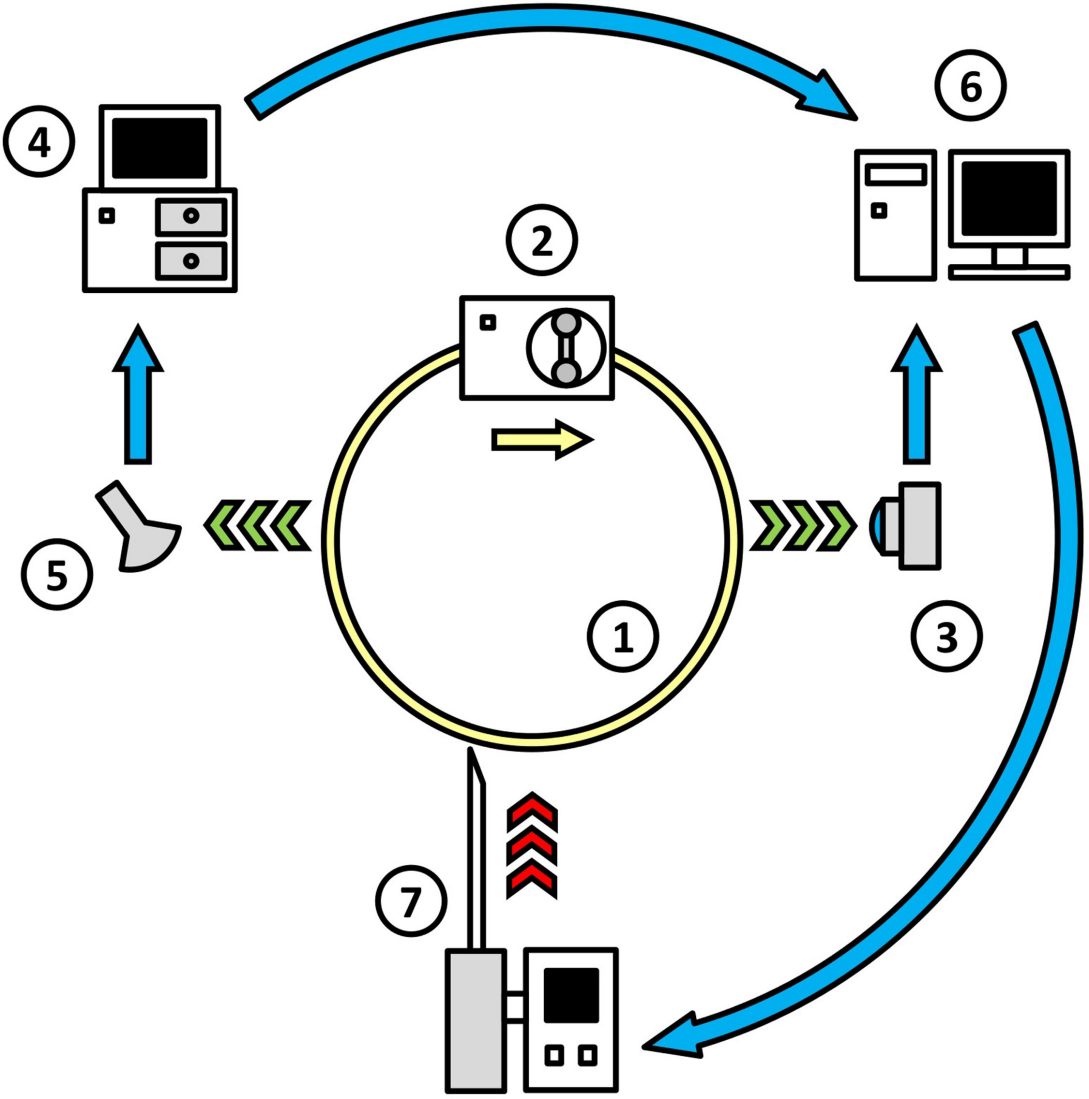
The layout of the experimental set-up. 1 –system of transparent flexible silicone tubes; 2 –peristaltic pump; 3 –digital camcorder; 4 –ultrasonic scanner; 5 –ultrasonic sensor; 6 –personal computer; and 7 –automated drug injector.

The principal scheme of the experimental setup is shown in Fig 1. A closed system of flexible transparent silicone tubes (1 in Fig 1) was filled with either blood or blood plasma. The inner diameter of the tube was 4 mm, and the total volume of the experimental system was 18 ml. The flow of liquid in the system was generated by a peristaltic pump, Elpan type 372.1 (2 in Fig 1). The mean velocity of the flow was kept at a rate of 20 cm/sec (shear rate up to 400 s^-1^). The activators of coagulation were injected in flowing blood directly when experiment started. All experiments were performed at the room temperature, 24 ± 2°C.

In the experiments with blood plasma, the processes of coagulation and fibrinolysis were registered both optically and acoustically. In the experiments with whole blood, due to its optical opacity, the registration was conducted only through the acoustic channel. Optical registration was performed in the transmitted light with a digital camcorder, GoPro HERO 3 (Woodman Labs, Inc., USA) (3 in Fig 1). A macro lens with an optical power of 21 diopters was used to focus the camcorder on the tube. The tube was held within the focal plane of the camera by a special screw clamp. The same screw clamp was used to create an area of local narrowing in the tube, beyond which a stagnation zone appeared in the flow. In several experiments, such a stagnation zone was created to facilitate the optical registration of fibrin microemboli, which form at an early stage of coagulation.

Acoustic registration was performed via an ultrasonic scanner Vingmed SD50 (Vingmed Sound; Norway) working in a Doppler mode at a frequency of 5 MHz (4 in Fig 1). To reduce the signal loss, the ultrasonic sensor (5 in Fig 1) together with a section of the tube system, was immersed in a bath filled with degassed water. The data from the optical and acoustical registration were recorded on a personal computer (6 in Fig 1).

A custom automated drug-injector (7 in Fig 1) was designed to perform the infusion of fibrinolytic drugs into the experimental system. The injector was connected to the computer via a Bluetooth channel. Following a signal from the computer, the injector delivered a fibrinolytic agent into the system in a precisely controlled and reproducible manner. The injection was performed gradually over 6 seconds, which was roughly equal to the turnover time of the liquid within the experimental system.

A special computer program was written in Python for real-time data analysis and the control of drug-injector operations. The Doppler shift of the ultrasonic signal was transferred from the scanner to the computer, digitized in a format of 44100 Hz, 16 bit and subjected to filtration by a second-order Butterworth filter with a passband from 200 to 1600 Hz [30]. Subsequently, the modulus of the amplitude of the filtered acoustic signal was averaged for 2-second time intervals. This value, indicated below as the averaged modulus of amplitude (AMA), was used for the monitoring of blood coagulation and fibrinolysis in the system. Upon the increase in AMA above a certain threshold, the program sent a command signal to the drug-injector to perform the injection. The threshold value of AMA was defined basing on a series of preliminary experiments as the background level of AMA in the beginning of the experiment multiplied by a predefined coefficient (equal to 2 for blood plasma and 1.3 for whole blood).

### Calculation of fibrinolysis efficiency index

The efficiency of fibrinolytic processes was assessed basing on the data from the acoustic registration after the end of each experiment. The area between the upper and the lower envelopes of the AMA curve was calculated for a time period of 60 minutes after the registration of the coagulation onset. This value calculated for the particular experiment was denoted as *S*_*exp*_, while *S*_*ref*_ stands for the respective value calculated for a reference experiment with the blood (blood plasma) of the same donor, but with normal saline instead of a fibrinolytic drug injected. Finally, fibrinolysis efficiency index (FEI) was calculated with the following formula:

$$FEI=1-\frac{S_{exp}}{S_{ref}}$$

It should be noted that *S*_*exp*_ is proportional to the integrated intensity of the acoustic signal reflected by macroscopic clots in the system during the experiment. The faster the dissolution of fibrin clots, the smaller the value of *S*_*exp*_. Accordingly, FEI tends to one in cases of the immediate dissolution of all fibrin clots and is close to zero in cases of the complete absence of lysis in the experimental system.

## Results

### Changes in the acoustic signal caused by the development of coagulation processes

**Fig 2:**
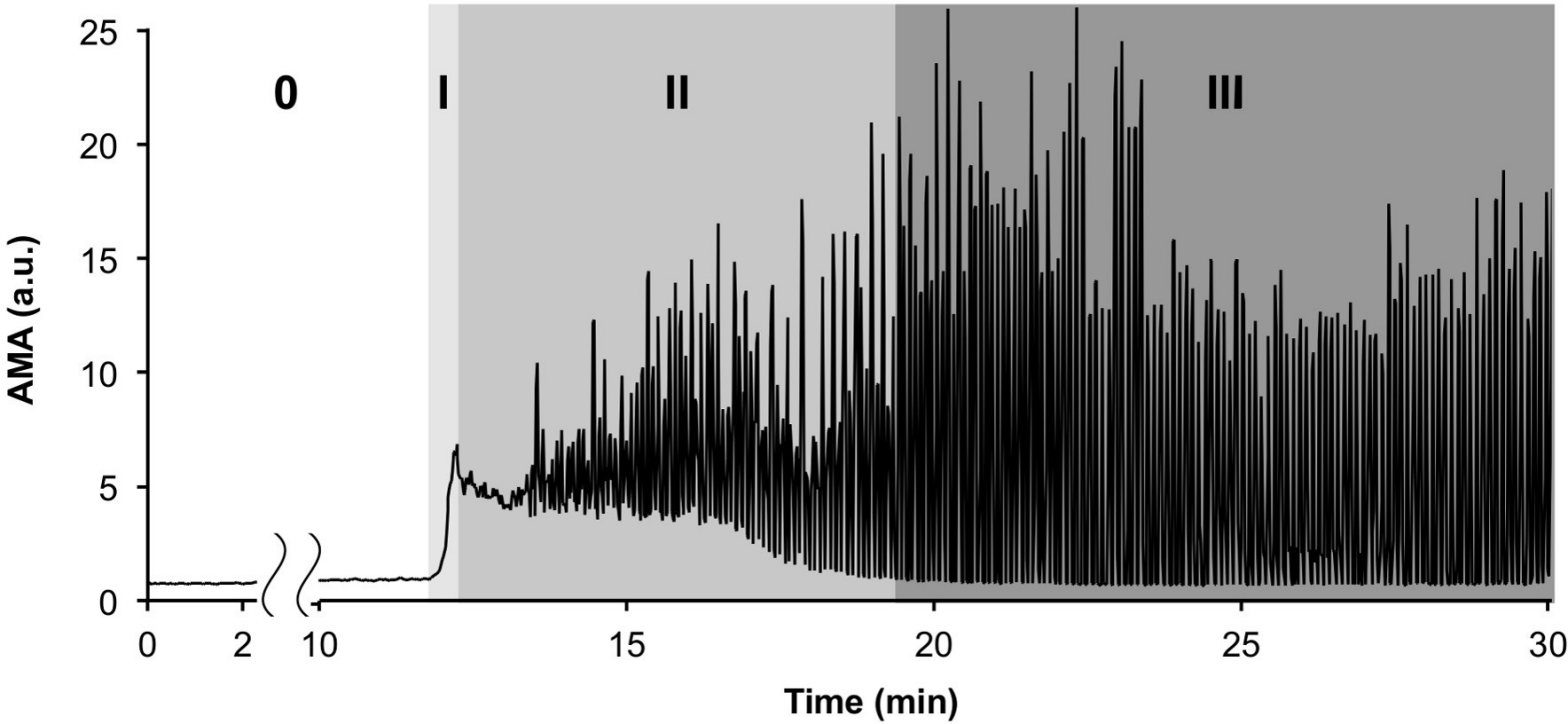
A typical time course of AMA (averaged modulus of amplitude of the acoustic signal) changes during coagulation in blood plasma. The four successive characteristic stages of the process are marked with numbers (0, I, II, III).

In the experiments with blood plasma, the changes in the acoustic signal were found to be correlated with the coagulation processes that were detected optically. The time course of AMA changes during a typical experiment with blood plasma is shown in Fig 2. The four distinct characteristic stages of the coagulation processes observed in all experiments are marked in Fig 2.

Stage “0” in Fig 2 corresponds to the lag phase that precedes the appearance of the first optically detectable fibrin microemboli in the system. This stage lasts for 10–20 minutes after the addition of coagulation activator to the plasma. The AMA value during this stage remained practically unchanged, and its fluctuations did not exceed 15% of the initial background level.

After the lag phase, the rapid formation of multiple fibrin microemboli in the flow begins (stage “I” in Fig 2). The movement of microemboli in the flow at this stage visually resembles a snow-storm. The increase in the amount of microemboli in the flow was accompanied by a drastic increase in the intensity of the reflected ultrasonic signal. A four-fold to six-fold increase in the AMA took place.

At 30–60 seconds following the appearance of the first microemboli, fibrin flakes that were several millimeters in size were formed in the system. During the following few minutes, gradual formation of larger aggregates took place in the system (stage “II” in Fig 2). This process was accompanied by the appearance of AMA oscillations, which were caused by single aggregates of various sizes passing in front of the ultrasonic sensor. The increase in the size of single clots led to the amplification of AMA oscillations at this stage.

Eventually, the mutual aggregation of fibrin flakes and microemboli led to the formation of several large macroscopic clots (stage “III” in Fig 2). The decrease in the number of clots in the system was accompanied by a decline in the lower envelope of the AMA plot. When the lower envelope of the AMA reached the initial background level of the AMA, no more sound-reflecting micro-aggregates remained in the flow. By that time, only several large clots remained in the system, causing large-amplitude oscillations in the AMA. These macroscopic clots were up to 10 cm in length and, in some experiments, were capable of occluding the vessel lumen completely, blocking flow.

Sample clips of a video recording of the coagulation process in blood plasma, representing all four characteristic stages, can be seen in S1 Video (see also S1 Fig). The video sequence is accompanied by a corresponding graph of AMA versus time. The Doppler shift in the ultrasonic signal is given as a soundtrack of this video record, enabling the coagulation processes developing in the system to literally be heard “with the naked ear”.

### Acoustic registration of drug-induced fibrinolysis

**Fig 3:**
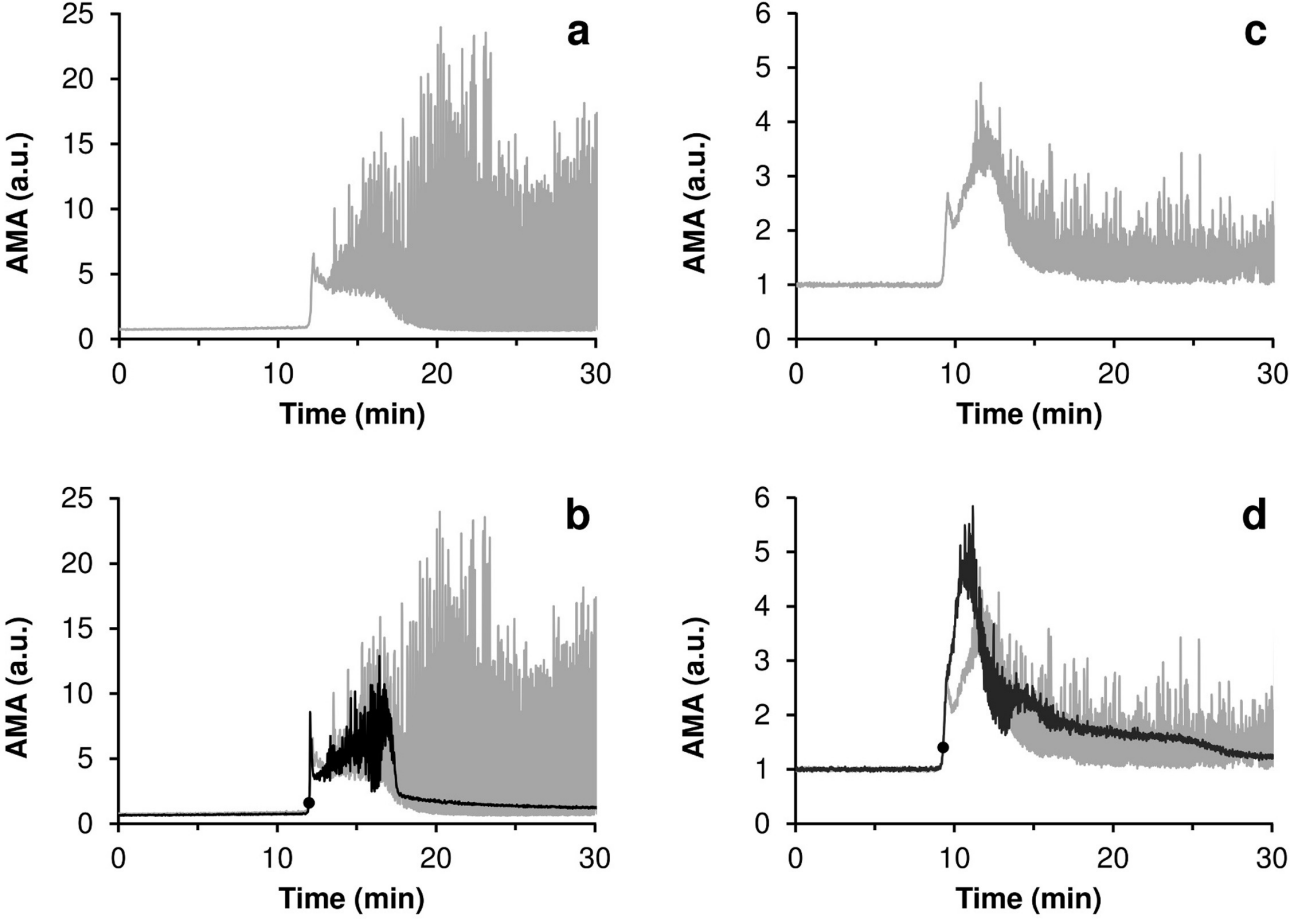
Typical curves of AMA (averaged modulus of amplitude of the acoustic signal) versus time for the experiments with a complete lysis of all macroscopic clots (black) in comparison with the reference curves obtained for the experiments in which no fibrinolytic drug was injected (gray). The moments of the injection of streptokinase are marked on the graphs with bold round markers. (a,b)–experiments with blood plasma, final concentration of streptokinase– 150 IU/ml; (c,d)–experiments with whole blood, final concentration of streptokinase– 600 IU/ml.

In Fig 3, the typical curves of AMA versus time for the experiments with fibrinolytic drug injections are presented in comparison with the reference curves obtained for the experiments in which no fibrinolytic drug was injected. In all cases final concentrations of fibrinolytics in the system after injection are indicated. The graphs for the experiments with blood plasma are given in Fig 3A and 3B, and those for the experiments with whole blood, in Fig 3C and 3D. It has been established that lysis of fibrin clots is reflected in corresponding changes in the acoustic signal. АМА represents total reflection of ultrasonic signal by fibrin clots present in the flow, i.e. macroscopic and microscopic clots. Dissolution of macroscopic clots is reflected in decrease of AMA oscillations. While dissolution of micro-clots is manifested by decrease of the lower envelope of AMA curve (see S1 Text for details).

The data presented indicate that the injection of a fibrinolytic drug at the initial stage of the coagulation process can prevent the formation of large clots in the experimental system. Moreover, it can be seen from Fig 3 that the practically complete lysis of all fibrin aggregates occurred in 5–7 minutes after injection of the fibrinolytic agent. Similar results were obtained for all coagulation activators used (see S2 Text). Sample clips of a video recording of the fibrinolysis process for an experiment with blood plasma are presented in S2 Video.

### Acoustic evaluation of the efficiency of fibrinolysis

The efficiency of fibrinolysis turned out to depend strongly on both the concentration of the fibrinolytic agent and on the moment of its injection. The best lysis was observed when the fibrinolytic agent was injected at the initial stage of the coagulation process (stage “I” in Fig 2).

To investigate the dependence of the efficiency of fibrinolysis on the concentration of the fibrinolytic drug, several series of experiments with identical drug injection timing were performed. The drug was injected immediately after the acoustic registration of the coagulation onset, as soon as the AMA exceeded the preset threshold level.

**Fig 4:**
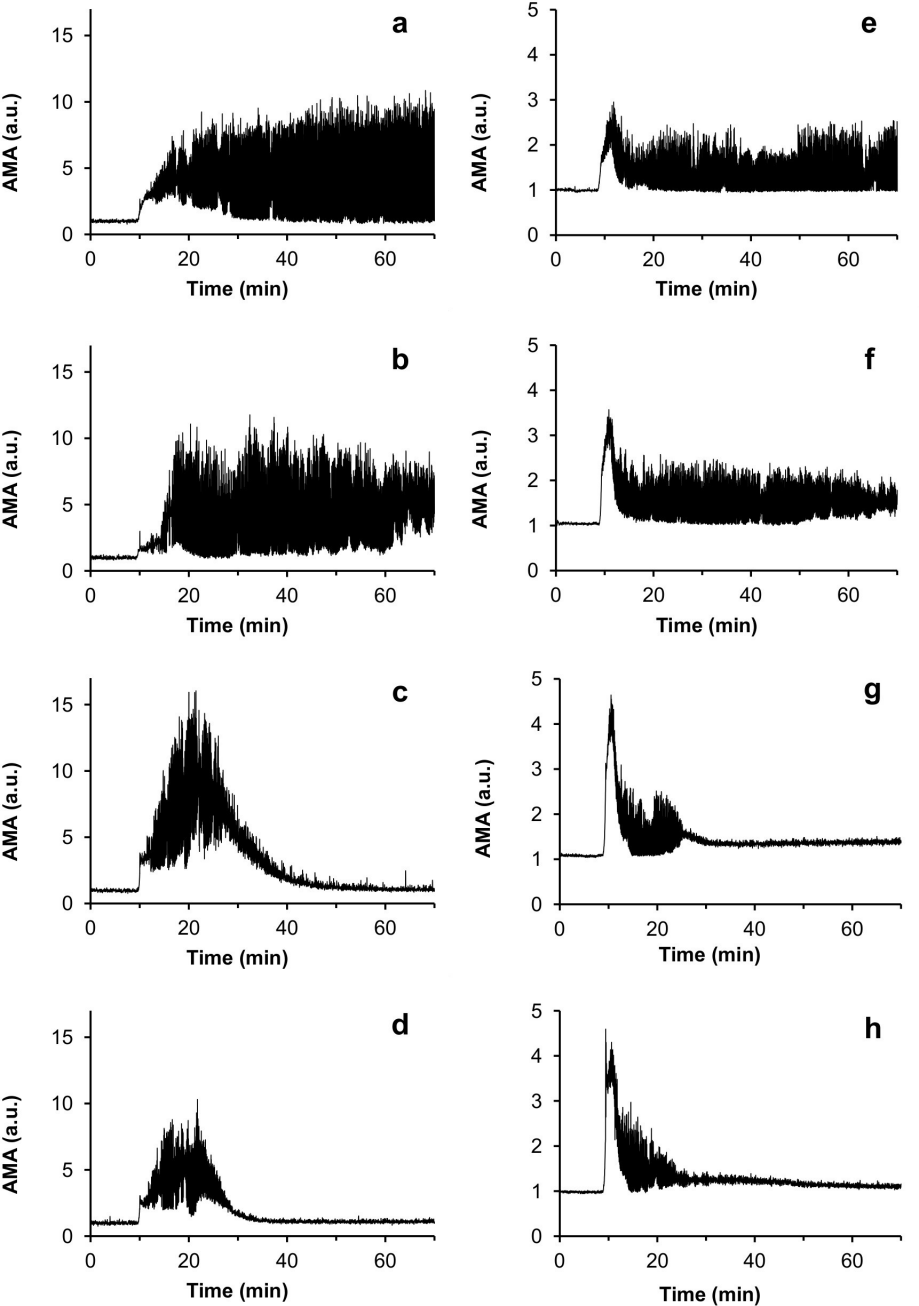
Curves of AMA (averaged modulus of amplitude of the acoustic signal) as a function of time for the experiments with different concentrations of urokinase injected. (a,b,c,d)–sets of experiments with blood plasma; (e,f,g,h)–sets of experiments with whole blood. The urokinase concentrations used were as follows: (a,e)–reference experiments, normal saline was injected instead of a fibrinolytic drug; (b,f)– 200 IU/ml; (c,g)– 625 IU/ml; (d,h) – 1250 IU/ml.

The curves showing AMA as a function of time for the experiments testing a series of varying concentrations of urokinase are presented in Fig 4. It is evident that the smaller the dose of fibrinolytic agent, the slower the dissolution of the fibrin clots. At urokinase concentrations of 50 IU/ml and lower, no fibrinolysis was observed during the 90-minute experimental period. The time course of AMA changes in these experiments was practically identical to that observed in the reference experiments, in which normal saline, instead of a fibrinolytic drug, was injected.

**Fig 5:**
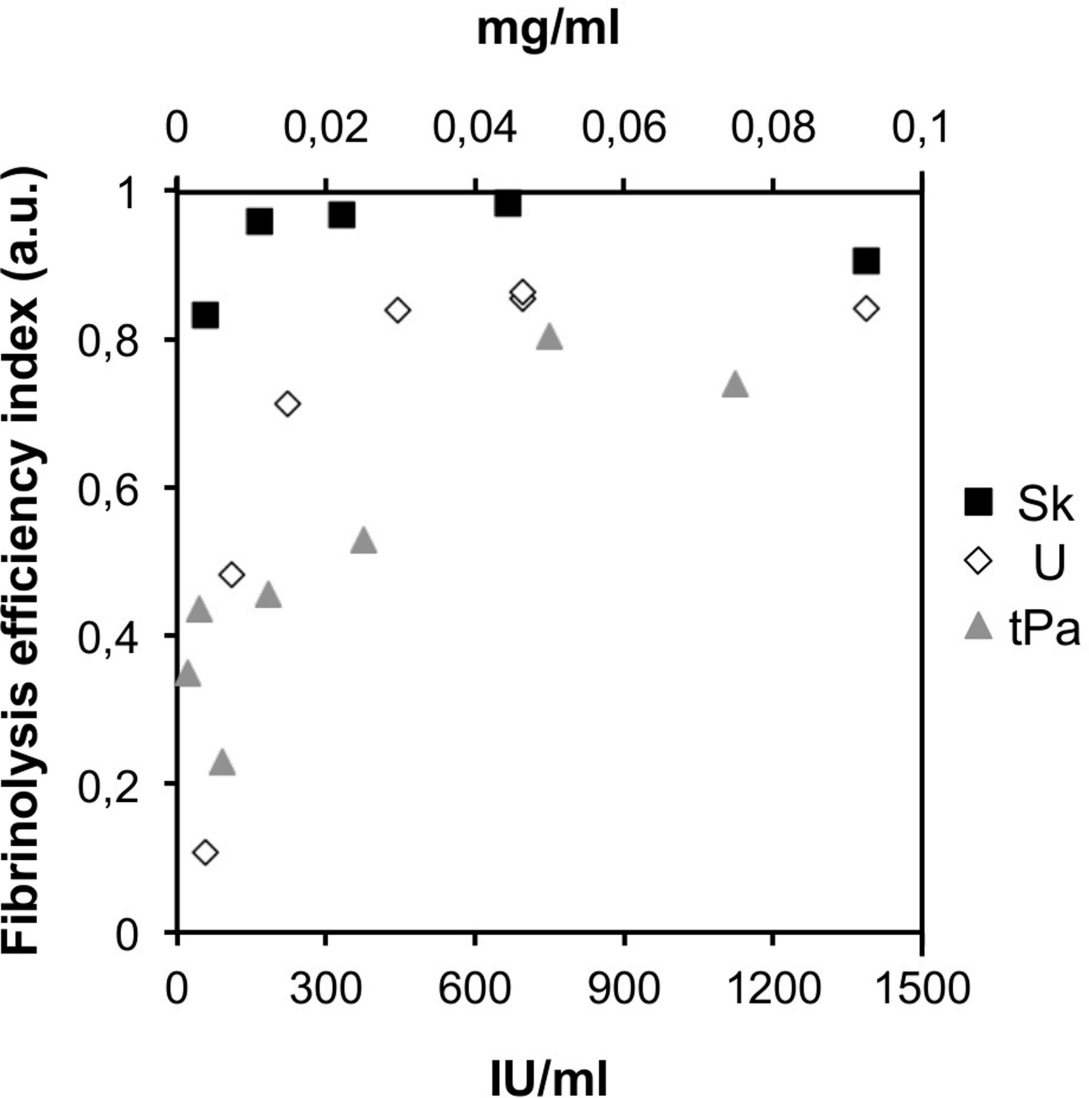
The dependences of FEI (fibrinolysis efficiency index) on the concentrations of the fibrinolytic agents used. The data for streptokinase are marked by black squares; the data for urokinase, by white diamonds; and the data for tissue-type plasminogen activator, by gray triangles. The concentrations for streptokinase and urokinase are indicated in IU/ml at the lower scalebar of the plot; the concentrations of t-PA are indicated in mg/ml at the upper scalebar of the plot.

To qualitatively compare the efficiency of fibrinolysis in different experiments, we introduced a special **fibrinolysis efficiency index (FEI)** (see “Materials and methods”). The dependences of FEI on the concentrations of the fibrinolytic agents used are presented in Fig 5. All data presented in Fig 5 correspond to the experiments with an injection of a fibrinolytic agent at the initial stage of the coagulation process. The sets of experimental points presented for each drug were obtained in a series of experiments using blood plasma from the same donor that was obtained on the same day.

### Increase in fibrinolytic resistance of fibrin clots during their formation

The rate of dissolution of the fibrin aggregates in the experimental system substantially depended on the time delay of fibrinolytic drug injection after the registration of coagulation onset. When the fibrinolytic agent was injected at the initial stage of coagulation (stage “I” in Fig 2), the complete dissolution of all of the clots in the system occurred in the following 5–15 minutes. However, the same concentration of a fibrinolytic drug may fail to cause any detectable lysis at all in cases where the injection was performed with a time delay of only several minutes after the appearance of the primary microemboli in the flow.

The dependence of the efficiency of fibrinolysis on the time delay of the injection was studied in detail with the aid of an automated drug injector. Several series of experiments were carried out in which the same dose of fibrinolytic drug was injected with different time delays after the registration of the appearance of first microemboli in the flow. The fibrinolytic injection was conducted either immediately after the AMA value doubly exceeded its initial background level or with a delay of 30, 60, 90, 120, 180 and 300 seconds.

**Fig 6:**
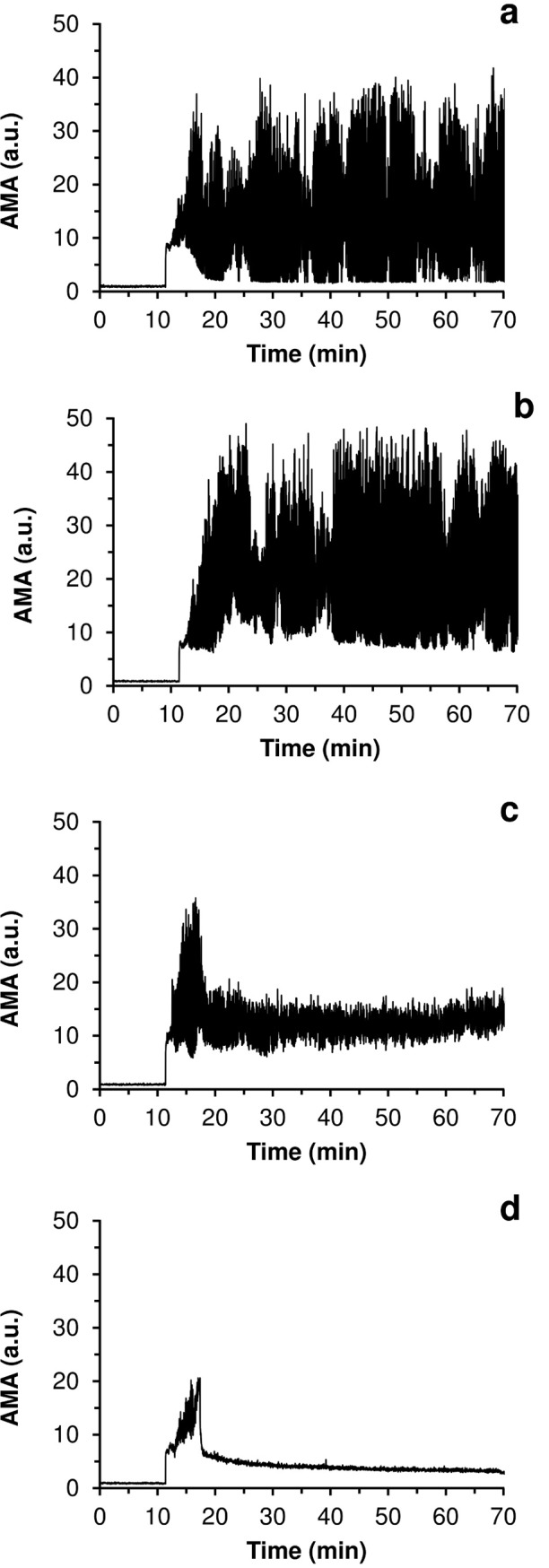
Curves of AMA versus time for an experimental series with different time delays of drug injection. a–control experiment with no fibrinolytic drug injected; b–injection delay of 300 seconds; c—injection delay of 60 seconds; d–injection no delay. A concentration of streptokinase was 250 IU/ml.

**Fig 7:**
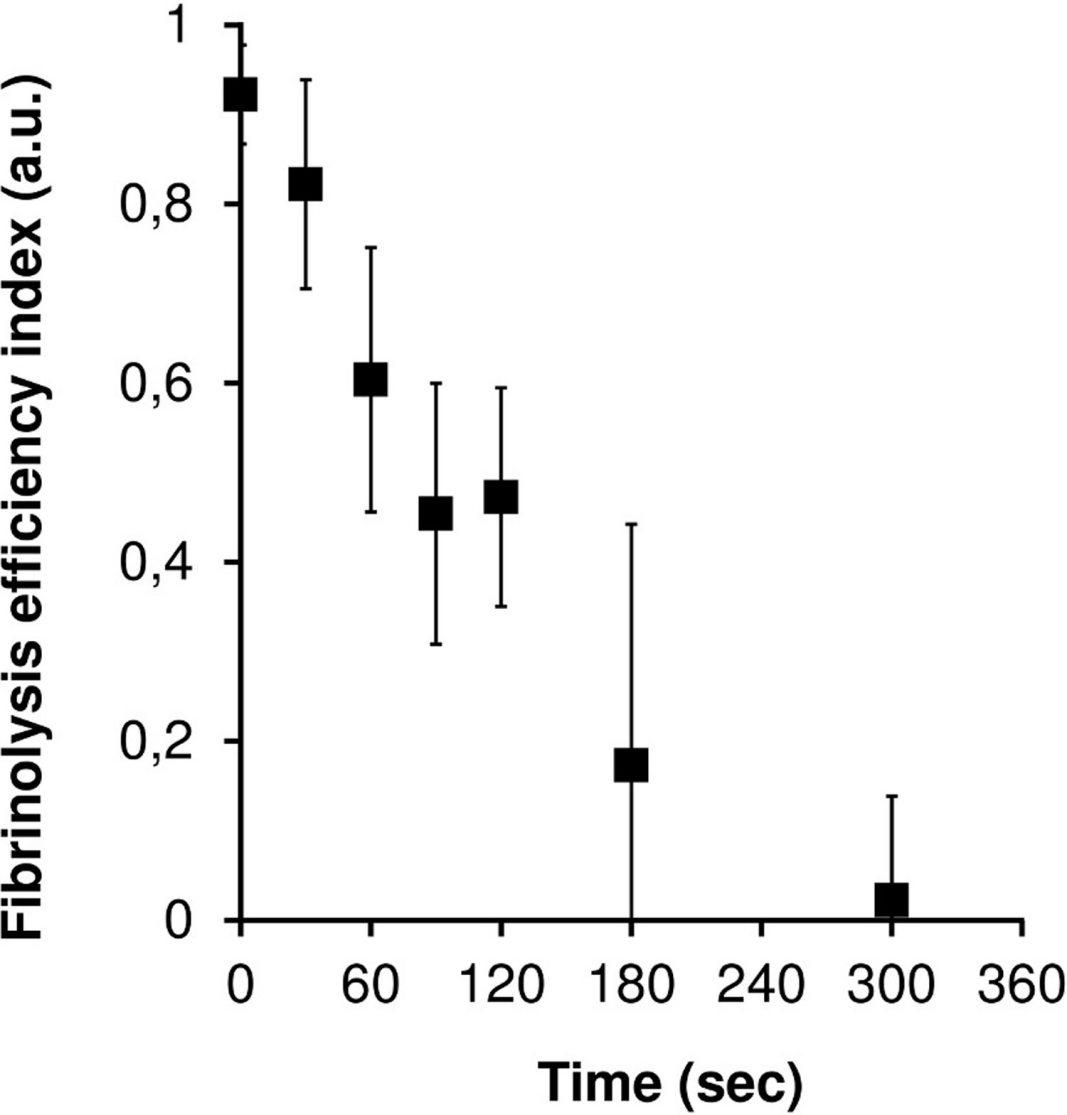
The dependence of FEI on the delay time of the fibrinolytic drug injection. The dependence was established on the basis of 6 series of experiments with blood plasma from different donors. The concentration of streptokinase injected was constant in all the experiments and was equal to 250 IU/ml.

The curves of AMA versus time for a series of experiments with different time delays of drug injection are presented in Fig 6. Fig 7 shows the dependence of FEI on the delay time of the fibrinolytic drug injection for 6 series of experiments with plasma samples of different donors. It can be seen that a delay in the injection of more than 30 seconds after the registration of the first microemboli leads to a significant decrease in the efficiency of fibrinolysis. In cases where the injection delay was 5 minutes or more, practically no lysis was observed.

A similar effect was observed in the experiments with urokinase and tissue-type plasminogen activator. An injection of a fibrinolytic drug during stage “I” led to fast and effective fibrinolysis, while at the beginning of stage “II”, it led to far less pronounced lysis, and at the end of stage “II” or during stage “III”, it caused practically no lysis at all. Thus, it can be concluded that the fibrinolytic resistance of clots increases drastically during the first 5 minutes of their formation.

## Discussion

Presently, ultrasonic methods are already used rather widely in the field of thrombosis and hemostasis, for instance, in the diagnostics of deep vein thrombosis [31, 32], the detection of thrombi in the left atrial appendage [33] and the monitoring of intravascular emboli [34]. Taking into account the recent achievements in the development of implantable ultrasonic sensors [35] it seems quite likely that, eventually, an ultrasonic technique for the monitoring of blood coagulation and thrombi formation inside the human body will be created.

In our previous works, we have shown the applicability of ultrasonic methods for the non-invasive registration of coagulation processes occurring under intensive blood flow conditions [24, 25]. Further development of these methods seems to be very promising because they may enable coagulation monitoring in the areas of the vascular system where thrombus formation poses the greatest threat to the patient’s life and health, specifically, the large vessels of heart and brain.

In the present study, it has been shown that ultrasonic methods enable the registration of coagulation processes at the stage when timely pharmacological intervention can still prevent the formation of macroscopic clots in the experimental system. Thus, it was shown that real-time ultrasonic registration of coagulation processes, in principle, provides the facility to control thrombi formation.

The results presented in this paper may open prospects for creating portable or even implantable devices, which would be somewhat similar to insulin pumps currently used in clinical practice [36]. By means of ultrasound, such a device could provide not only the monitoring of blood clotting and fibrinolysis, but also active control of these processes. The miniature portable injector with several Doppler sensors on critical human arteries could timely inject fibrinolytics directly at the early stage of clotting when hemostasis could be corrected faster and more efficiently.

In our experiments the resistance of clots to fibrinolysis increased drastically in the first few minutes of clots formation. The increase in the resistance of clots to the action of fibrinolytic agents with time is well known in clinical practice [28, 37]. Although our results show a similar trend to that of clinical observations, the time period within which the clots remained sensitive to the action of fibrinolytic agents turned out to be at least ten fold shorter in our experiments. The particular mechanisms underlying such a rapid increase in the fibrinolytic resistance of the clots are still unclear. However, it may be assumed that the effect observed in our work is the result of chemical stabilization of the clots on one hand [38], and on the other hand, changes in the structure of the clots, leading to a decrease in the permeation of fibrinolysis activators to the inner areas of the clots [39].

Concerning chemical stabilization of fibrin clots it is generally known that the action of coagulation factor XIII [38] and thrombin activatable fibrinolysis inhibitor (TAFI) [40] substantially increase the fibrinolytic resistance of forming clots. Both of the factors are converted to their active forms by thrombin. Activated factor XIII restrain fibrinolysis by covalent linking of α2-antiplasmin to fibrin [41] as well as by cross-linking of α- and/or γ-chains of fibrin [42]. Activated TAFI down-regulates fibrinolysis by removal of C-terminal lysines from fibrin, preventing in that way binding and activation of plasminogen [43].

Moreover it is worth to mention that blood flow itself can influence fibrinolytic resistance of forming clots in a bidirectional manner. On one hand flow influences the structure of the fibrin network [44, 45], making it more dense and less permeable to lytic agents, thus impeding the fibrinolytic process [46]. On the other hand, the flow substantially influences the character of the mass transfer inside the clot and near its surface, thus accelerating the fibrinolytic dissolution of the clots [47, 48].

Keeping this in mind, within the present work, it was essential to create the experimental conditions of flow to mimic thrombi formation taking place in large arteries. The experimental scheme chosen for this purpose is in a way, analogous to the well-known Chandler system [49], which is widely used up to date to create artificial clots mimicking arterial thrombi [50, 51]. Despite some differences in the setups, the development of coagulation processes in our experimental system was, in many aspects, similar to that observed in a classical Chandler system. For instance, the stage of multiple microemboli formation in the flow, resembling a “snow-storm”, which was observed in our experiments, was previously described for the Chandler system in experiments by McNicol et al [52].

Of course no *in vitro* experimental system could completely reproduce *in vivo* formation of arterial thrombi. Certainly, further *in vivo* investigations are required to answer a general question: whether the monitoring of the early stages of blood coagulation can increase the real clinical facilities for the prevention of thrombotic complications. Until recently, practically all research on the ultrasonic registration of blood coagulation has been carried out with *in vitro* model systems [7–18, 23–25, 53]. A few novel studies in this research field that have employed *in vivo* experiments have been published just recently [20–22]. The small number of such works may be attributed to the necessity of the convergence of several branches of modern science to carry out this type of research. We hope that the present work will attract additional interest and attention among researchers to further address the problems of ultrasonic monitoring of blood coagulation *in vivo*.

## Supporting information

S1 Text Interpretation of acoustic signals reflected by plasma and whole blood. (PDF)

S2 Text Registration of coagulation onset initiated by different types of activators. (PDF)

S1 Video Coagulation under intensive flow conditions registered optically and acoustically. (AVI)

S2 Video Dissolution of fibrin clots induced by fibrinolytic drug injection. (AVI)

S1 Fig Frames from S1 Video. Acoustic channel is marked by blue frame. Optical channel is marked by red frame. Four successive characteristic stages of the coagulation process are presented as: (A)–lag phase, (B)–“snow-storm” phase, (C)–clots aggregation phase, (D)–macroscopic clot.
(TIF)

S2 Fig Frames from S2 Video. Acoustic channel is marked by blue frame. Optical channel is marked by red frame. Successive characteristic stages of the fibrinolysis process are presented as: (A)–large fibrin clot, (B)–partially lysed clot fragments, (C)–final stage of clot dissolution.
(TIF)

S3 Fig AMA curves for experiments with whole blood (a) and blood plasma (b) plotted in the same scale. Both curves are normalized on the initial signal level observed in the experiment with blood plasma.
(TIFF)

S4 Fig Curves of AMA (averaged modulus of amplitude of the acoustic signal) for the experiments with different coagulation activators used. (a, d)– 50 μl of 1% kaolin suspension; (b, e)– 600 μl of 10% calcium chloride solution; (c, f)– 50 μl of thromboplastin solution, diluted by 12 times with normal saline; (a, b, c)–experiments with no fibrinolytic agent injected; (d, e, f)—experiments with 1250 IU/ml urokinase injected. All experiments were performed with plasma of the same donor collected at the same day.
(TIFF)
