# Supplementary material for: Control of fibrinolytic drug injection via real-time ultrasonic monitoring of blood coagulation
Source: PLoS One. 2019 Feb 27;14(2):e0211646. doi: 10.1371/journal.pone.0211646 (PMC6392241; doi:10.1371/journal.pone.0211646)
Supplement: S1 Text — (PDF) [file pone.0211646.s001.pdf]

S1 Interpretation of acoustic signals reflected by plasma and whole blood

Reflection of ultrasonic signal in our experimental system takes place due to fibrin clots of several scales. Reflection of ultrasound by macroscopic clots (clot size >> acoustic wave length (λ)) could be described by the laws of geometric optics. Reflection from micro-clots (clot size << λ) could be described by Rayleigh scattering [S1.1]. For clots with size comparable with λ no general solution for scattering problem is known [S1.2].

The wave length in our experiments is approximately equal to:

$$\lambda \approx \frac{1500\ \mathrm{m/s}}{5 \times 10^{6}\ \mathrm{s}^{-1}} = 0.3\ \mathrm{mm}$$

During coagulation and subsequent fibrinolysis reflection of the signal occurs at all three scales mentioned above. Moreover during formation and lysis of clots not only their growth/dissolution take place, but also aggregation/fragmentation might occur [S1.3]. Some purely theoretical aspects of coagulation processes were considered earlier in [S1.4]. Never the less complete theoretical description of ultrasound reflection on forming clots seems to be not developed yet.

Due to the complexity of the process considered, in the present work we used simplified approach for ultrasonic signal interpretation. We assume that averaged modulus of amplitude of the acoustic signal (AMA) represented the total reflected acoustic signal by both micro- and macro-clots. To make conclusions about the time evolution of clots present in the system following points were taken into account:

1. Individual large clots passing the ultrasonic sensor corresponded to individual peaks in AMA signal. The oscillating part of AMA represents the reflection of the acoustic signal from

successively moving macroscopic clots. As the size of large clots decreases during lysis the oscillations of AMA diminish too. On the contrary, if activation of fibrinolysis is relatively weak temporary increase of the oscillatory part of AMA might occur at the beginning of lysis (see first 20 min in Figs 4b and 4c in comparison to Fig 4a). In these cases fragmentation of clots into macroscopic fragments prevails over the decrease of their mass due to lysis and a transitory increase of total scattering cross section is observed.

2. The part of reflecting signal generated by Rayleigh scattering on micro-clots contributed to the level of lower envelope of AMA curve. The decrease of the lower envelope of AMA could be caused either by aggregation of all the micro-clots with larger clots (in case of clotting see Figs 2, 3a, 4a, 6a) or by dissolution of micro-clots in case of fibrinolysis (see Figs 3b, 4c, 4d, 6d). In contrast fragmentation of large clots could increase amount of micro-clots, leading to transient increase in AMA lower envelope at the initial stages of clots' lysis (see Figs. 3d, 4c, 4g, 4h).

Considering experiments with whole blood it is necessary to take into account backscattering of ultrasonic signal by red blood cells. According to modern concepts, scattering occurs on density and compressibility fluctuations caused by random changes in the red blood cells concentration [S1.5]. Moreover several dynamic effects influence the reflection of ultrasound by red cells (including aggregation) modulated by shear flow [S1.6, S1.7].

In S3 Fig AMA curves obtained for whole blood and blood plasma are presented. It can be seen that signal reflected by red blood cells partially masks the coagulation process. However major results obtained in the experiments with blood plasma remained valid for whole blood. Particularly:

- The ultrasonic method used enables the reliable registration of blood coagulation and following fibrinolytic dissolution of clots.

- An immediate injection of a fibrinolytic drug after the ultrasonic registration of the onset of coagulation is able to prevent the formation of large clots in the experimental system.

S1 References:

S1.1. Strutt, J. W. The theory of sound, vol. 2, 2nd edn. London, UK: Macmillan; 1896. p. 534.

S1.2. Ishimaru A. Wave Propagation and Scattering in Random Media, Wiley-IEEE Press; 1999. p. 600.

S1.3. Guria GT, Herrero MA, Zlobina KE. Ultrasound detection of externally induced microthrombi cloud formation: a theoretical study. J Eng Math. 2009;66(1-3):293-310.

S1.4. Lushnikov AA, Kulmala M. Nucleation burst in a coagulating system. Phys Rev E Stat Phys Plasmas Fluids Relat Interdiscip Topics. 2000;62(4, Pt A):4932-9.

S1.5. Hill CR, Bamber JC, ter Haar GR, editors. Physical principles of medical ultrasonics, 2nd Edition. John Wiley & Sons, Ltd; 2005.

S1.6. Cloutier G, Qin Z. Ultrasound backscattering from non-aggregating and aggregating erythrocytes-a review. Biorheology. 1997;34(6):443-470.

S1.7. Paeng D-G, Nam K-H. Ultrasonic Visualization of Dynamic Behavior of Red Blood Cells in Flowing Blood. Journal of Visualization. 2009;12(4):295-306.
