# Supplementary material for: Control of fibrinolytic drug injection via real-time ultrasonic monitoring of blood coagulation
Source: PLoS One. 2019 Feb 27;14(2):e0211646. doi: 10.1371/journal.pone.0211646 (PMC6392241; doi:10.1371/journal.pone.0211646)
Supplement: S2 Text — (PDF) [file pone.0211646.s002.pdf]

S2 Registration of coagulation onset initiated by different types of activators

To determine whether the use of different methods of activation of coagulation system affects the ability of early registration of coagulation special series of experiments was performed. In these experiments three different types of coagulation activators were tested: 50 µl of 1% kaolin suspension (NPO-Renam, Russia), 600 µl of 10% calcium chloride solution (Mapichem AG, Switzerland) and 50 µl of thromboplastin solution, diluted by 12 times with normal saline (NPO-Renam, Russia). Prior to infusion of each of these activators an injection of 300 µl of 10% calcium chloride solution was performed. This injection alone was insufficient for activation of coagulation in the experimental system, partially reconstituting calcium consumed by citrate phosphate dextrose (CPD). Control experiments have shown that after such a reconstitution plasma doesn't clot at least for 4 hours.

Regardless of the type of activator used, the initial stage of coagulation was revealed as a rapid increase in AMA (averaged modulus of amplitude of the acoustic signal) (see S4 Fig (a,b,c)). This fact allowed us to use the automated drug-injector to input fibrinolytic agent into the system precisely at the beginning of coagulation process. Injection of 1250 IU/ml urokinase (Urokinase, Medac GmbH, Germany) was performed as described in "Materials and methods" section. AMA curves for experiments with activation of fibrinolysis are shown in S4 Fig (d,e,f). It can be seen from S4 Fig that regardless of the type of coagulation activator the injection of a fibrinolytic drug at the initial stage of the coagulation allowed to stop the formation of large clots and dissolve all fibrin clots in the system.
