# Supplementary figures and images for: Control of fibrinolytic drug injection via real-time ultrasonic monitoring of blood coagulation
Source: PLoS One. 2019 Feb 27;14(2):e0211646. doi: 10.1371/journal.pone.0211646 (PMC6392241; doi:10.1371/journal.pone.0211646)

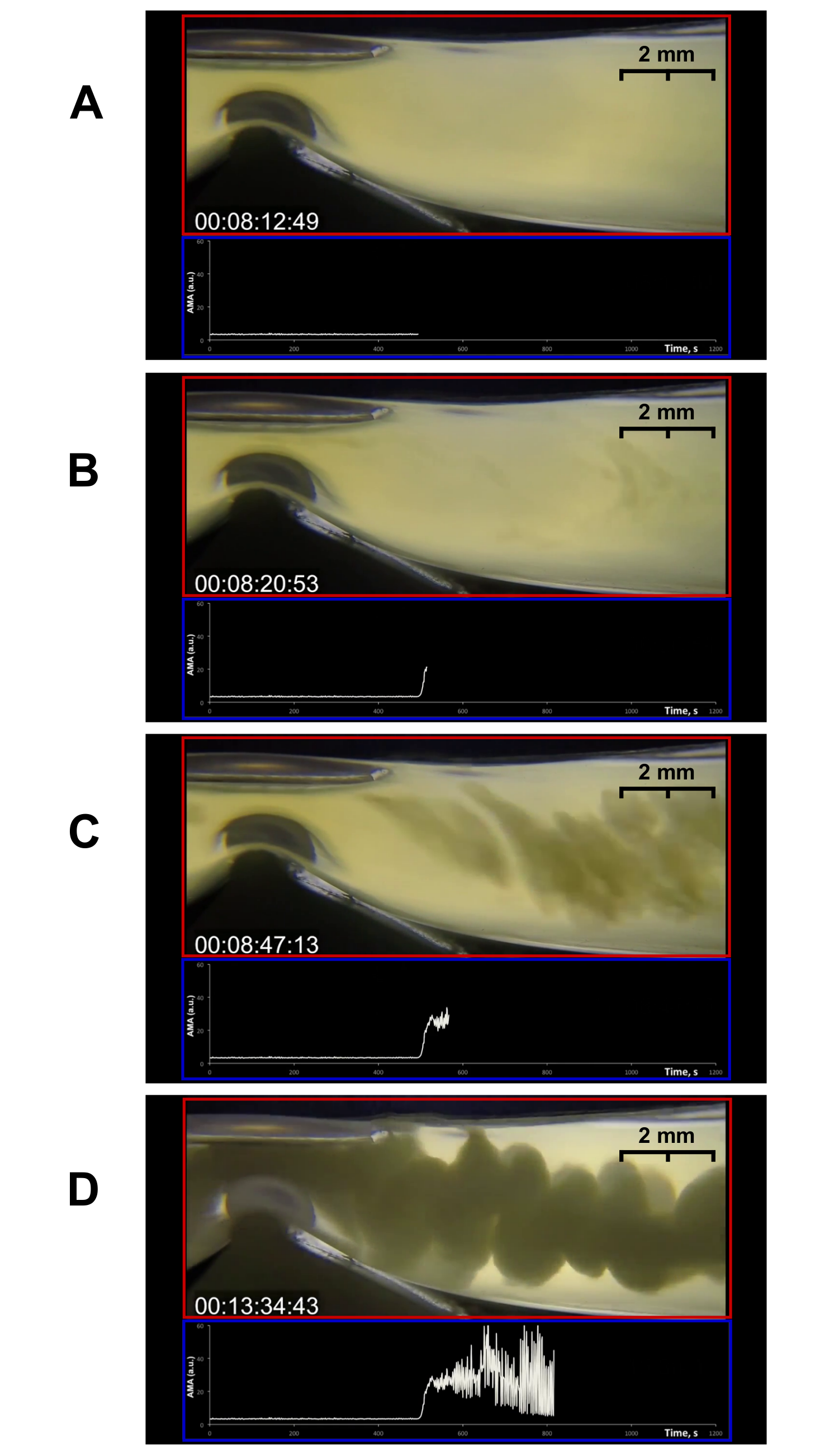

Supplement: S1 Fig — Acoustic channel is marked by blue frame. Optical channel is marked by red frame. Four successive characteristic stages of the coagulation process are presented as: (A)–lag phase, (B)–“snow-storm” phase, (C)–clots aggregation phase, (D)–macroscopic clot. (TIF) [file pone.0211646.s005.tif]

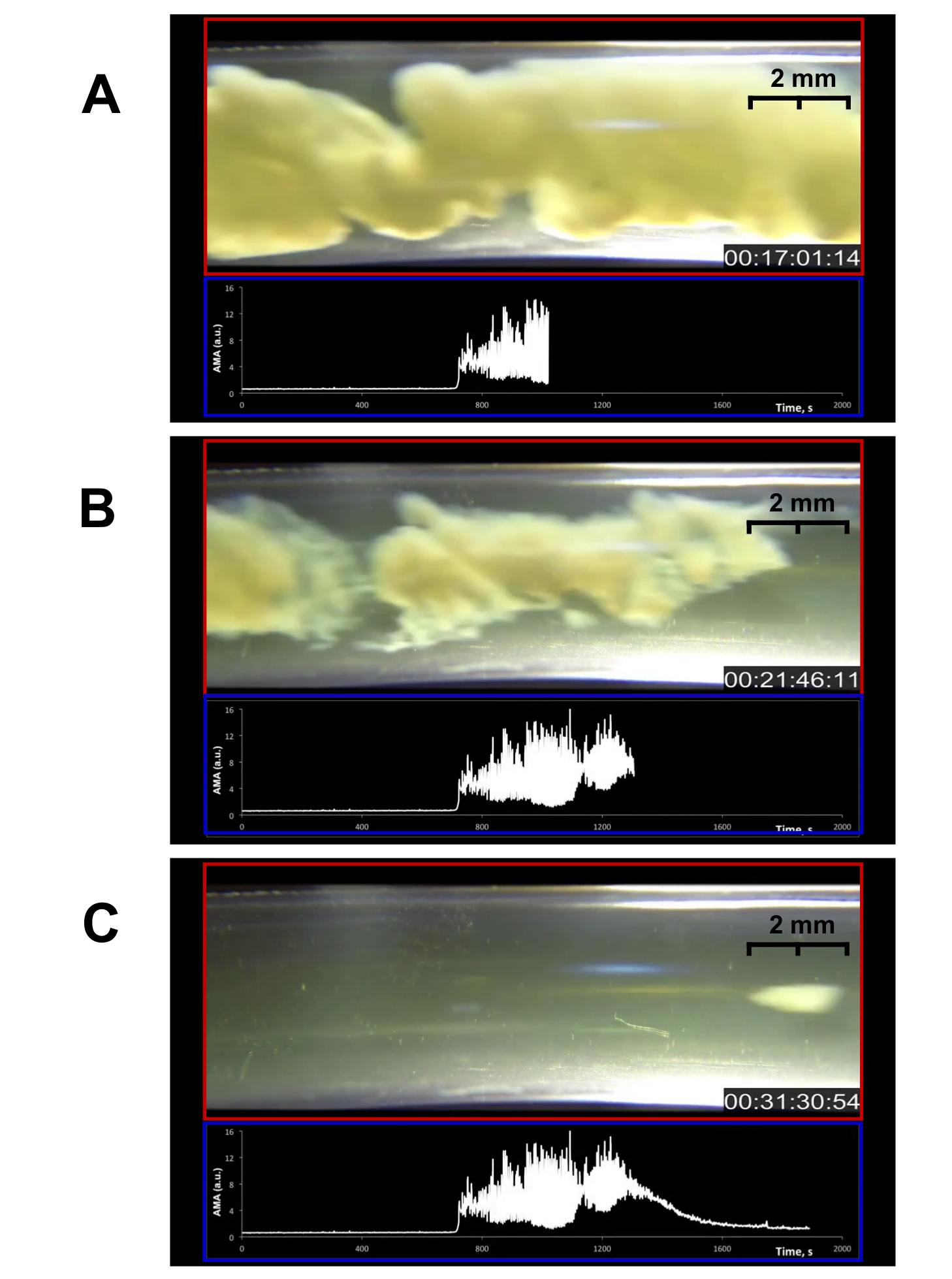

Supplement: S2 Fig — Acoustic channel is marked by blue frame. Optical channel is marked by red frame. Successive characteristic stages of the fibrinolysis process are presented as: (A)–large fibrin clot, (B)–partially lysed clot fragments, (C)–final stage of clot dissolution. (TIF) [file pone.0211646.s006.tif]

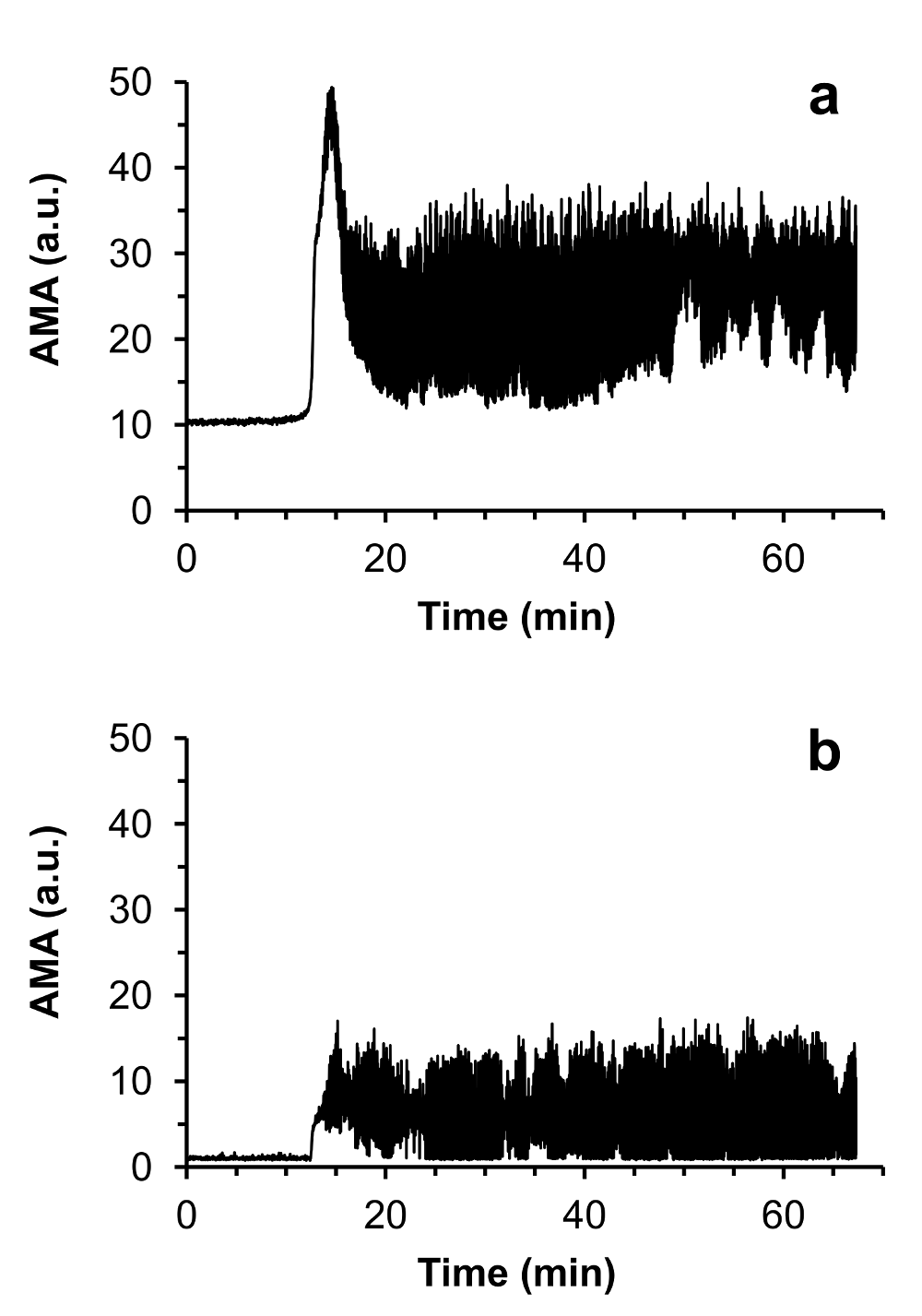

Supplement: S3 Fig — AMA curves for experiments with whole blood (a) and blood plasma (b) plotted in the same scale. Both curves are normalized on the initial signal level observed in the experiment with blood plasma. (TIFF) [file pone.0211646.s007.tiff]

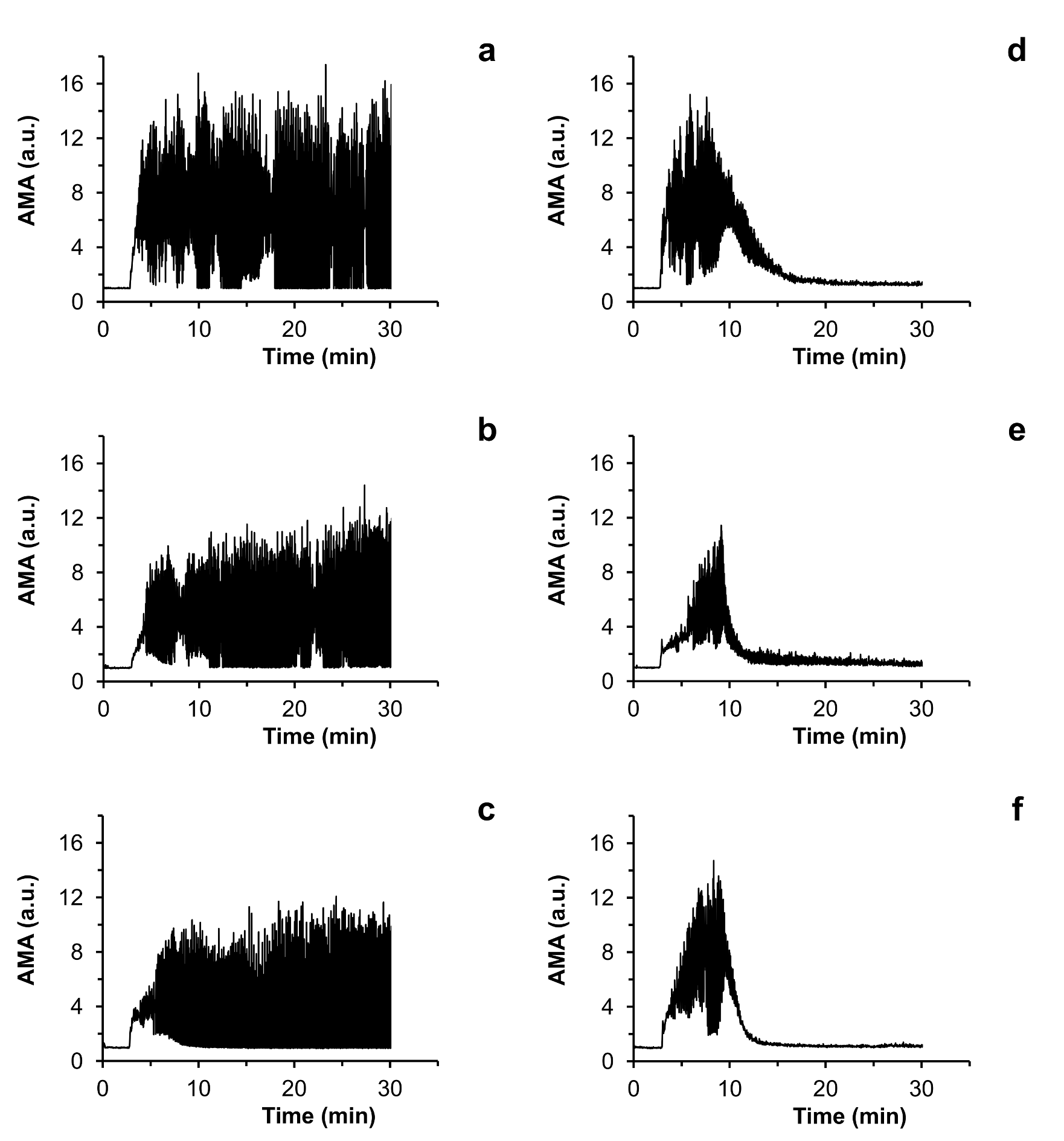

Supplement: S4 Fig — (a, d)– 50 μl of 1% kaolin suspension; (b, e)– 600 μl of 10% calcium chloride solution; (c, f)– 50 μl of thromboplastin solution, diluted by 12 times with normal saline; (a, b, c)–experiments with no fibrinolytic agent injected; (d, e, f)—experiments with 1250 IU/ml urokinase injected. All experiments were performed with plasma of the same donor collected at the same day. (TIFF) [file pone.0211646.s008.tiff]
